# Pathogenicity and Growth Conditions Modulate *Fonsecaea* Extracellular Vesicles’ Ability to Interact With Macrophages

**DOI:** 10.3389/fcimb.2022.879018

**Published:** 2022-06-09

**Authors:** Lucas de Oliveira Las-Casas, Clara Luna Freitas Marina, Raffael Júnio Araújo de Castro, Luísa Coutinho Coelho, Sônia Nair Báo, G. Sybren de Hoog, Vânia Aparecida Vicente, Larissa Fernandes, Anamelia Lorenzetti Bocca

**Affiliations:** ^1^ Department of Cell Biology, University of Brasília, Brasília, Brazil; ^2^ Department of Pathology, Federal University of Paraná, Curitiba, Brazil; ^3^ Center of Expertise in Mycology of Radboud, University Medical Center/Canisius Wilhelmina Hospital, Nijmegen, Netherlands; ^4^ Faculty of Ceilândia, University of Brasília, Brasília, Brazil

**Keywords:** chromoblastomycosis, extracellular vesicles, *Fonsecaea*, macrophages, muriform cells

## Abstract

Chromoblastomycosis (CBM) is a chronic cutaneous and subcutaneous mycosis caused by black, dimorphic, and filamentous fungi of the *Herpothrichiellaceae* family, such as species of the genus *Fonsecaea*. These fungi can switch between the saprophytic forms (conidia and hyphae) and the pathogenic form, the muriform cells (MCs), which is considered an essential mechanism for fungal virulence. Nearly all types of cells can produce membranous structures formed by a lipid bilayer that communicate extracellularly with other cells, known as “extracellular vesicles” (EVs), which may act as virulence factors, as observed for several species of pathogenic fungi. Our findings demonstrated for the first time that *F. pedrosoi, F. nubica*, and *F. erecta* produce EVs in response to nutritional conditions. The EVs varied in sterol and protein contents, size, and morphology. Moreover, the EVs induced different cytokine and nitric oxide release patterns by bone marrow-derived macrophages (BMDMs). The EVs activated IL-1β production, possibly acting as the first signal in inflammasome activation. Unlike the pathogenic species, the EVs isolated from *F. erecta* did not significantly stimulate TNF and IL-10 production in general. Overall, these results demonstrated that different species of *Fonsecaea* produce EVs capable of modulating pro- and anti-inflammatory cytokine and nitric oxide production by BMDMs and that growth conditions affected the immunomodulatory capacities of the EVs as well as their size, content, and morphology.

## Introduction

Chromoblastomycosis (CBM) is one of the most predominant cutaneous and subcutaneous infections encountered, especially in tropical and subtropical regions of the world (Attapattu, 1997; Bonifaz et al., 2001; Queiroz-Telles et al., 2009; Lu et al., 2013). CBM is a neglected disease by the World Health Organization (WHO, 2013). It is a chronic and granulomatous mycosis caused by a group of melanized and dimorphic fungi belonging to the *Herpotrichiellaceae* family (order *Chaetothyriales*). The disease is acquired through the traumatic inoculation of fungal propagules into the human tissue and can affect immunocompetent individuals (Mcginnis, 1938; Rubin et al., 1991; Marques et al., 2006; Guedes et al., 2010; Queiroz-Telles, 2015; Brito and Bittencourt, 2018). The most well-known etiological agent of CBM is *Fonsecaea pedrosoi*. However, species such as *F. monophora*, *F. nubica*, and *F. pugnacious* are important pathogens to humans (Santos et al., 2007; Najafzadeh et al., 2009; Najafzadeh et al., 2010; de Azevedo et al., 2015; Feng and de Hoog, 2017). All pathogenic species of the *Fonsecaea* genus are morphologically and genetically similar, but the disease has different clinical aspects and geographic distributions (Najafzadeh et al., 2010; Teixeira et al., 2017; Vicente et al., 2017). Clinically, *F. pedrosoi* and *F. nubica* are exclusively related to CBM, whereas *F. monophora* and *F. pugnacious* can infect different organs and are considered opportunistic pathogens (Santos et al., 2007; Najafzadeh et al., 2009; Najafzadeh et al., 2010). Except for *F. pedrosoi*, which is restricted to South and Central America, all other species have also been described in Asia, Africa, and Europe (Xi et al., 2009; Cateau et al., 2014; de Azevedo et al., 2015; Fransisca et al., 2017; Label et al., 2018; Rasamoelina et al., 2020).

An essential characteristic of CBM is the presence of highly melanized thick-walled cells, called sclerotic bodies or muriform cells (MCs), that are crucial for fungal persistence inside host tissues, protecting against environmental stresses (Alviano et al., 1992; López-Martínez and Méndez-Tovar, 2007; Feng and de Hoog, 2017; Dong et al., 2020). These cells are considered the key factor for *Fonsecaea* pathogenicity and are related to chronic disease. (Hamza et al., 2003; Salgado et al., 2010; Siqueira et al., 2017; Dong et al., 2020).

The capacity of *Fonsecaea* spp. to undergo the transition between the saprophytic (conidia and hyphae) and the parasitic (MCs) morphotypes is considered the main requirement for fungal persistence on the human tissue (Queiroz-Telles et al., 2017; Siqueira et al., 2017; Dong et al., 2018). The conidial and hyphal forms of *Fonsecaea* are frequently found in the environment and during the initial stages of CBM, which are marked by fungal inoculation, adhesion to the tissue, and proliferation (Queiroz-Telles, 2015; Siqueira et al., 2017; Dong et al., 2020). Once the fungus has infected the host, MCs become the predominant cells found within the granulomatous and suppurative centers of the lesions observed in CBM (Bonifaz et al., 2001; Minotto et al., 2001; Queiroz-Telles et al., 2017). Nutritional conditions, temperature, and stress factors, such as calcium deprivation and pH, are considered important factors that elicit fungal morphogenesis (Alviano et al., 1992; Mendoza et al., 1993; Silva et al., 2002; Santos et al., 2007). *In vitro*, highly nutritional medium (*e.g.*, Sabouraud Dextrose Broth and Potato Dextrose Broth) induces mainly conidia and hyphae formation (Alviano et al., 1992; Siqueira et al., 2017). In contrast, in conjunction with propranolol – a beta-adrenergic antagonist – supplementation, as proposed by Alviano et al. (1992), minimum medium conditions can promote MCs production that is morphologically similar to those obtained *in vivo* (Alviano et al., 1992; Silva et al., 2002; Dong et al., 2018). The molecular mechanisms and virulence factors related to CBM remain not-so-well-known, although it is clear that microenvironmental factors play essential roles on *Fonsecaea* morphological transition and survival inside the host.

Recently it has been shown that hyphae and MCs of *F. pedrosoi* are related to the intense pro-inflammatory response found in CBM. At the same time, conidia alone cannot induce the chronic phase of the disease in the animal model of infection nor stimulate the production of important cytokines such as TNF, IL-1β, and IL-6 *in vitro* (Siqueira et al., 2017). Furthermore, *F. pedrosoi* hyphae, but not conidia, are capable of promoting IL-1β secretion by macrophages and dendritic cells through NLRP3-caspase-1 inflammasome activation, a crucial mediator of the host immune response during fungal infections (Vanaja et al., 2015;  de Castro et al., 2017). Additionally, current research indicates that the early stages of *F. pedrosoi* infection in a self-healing murine CBM model are mediated mainly by Th17 response through Dectin-2 receptors (Siqueira et al., 2020). In contrast, a pro-inflammatory Th1 response profile becomes predominant in later stages of the disease, which is necessary to combat MCs and is associated with the healing of the lesion (Siqueira et al., 2020). Lastly, it was demonstrated that Treg cells are pivotal for fungal clearance, and deregulation of these cells is involved with improper host immune response (Siqueira et al., 2020).

Extracellular vesicles (EVs) are an embracing term to refer to membranous structures composed of a phospholipid bilayer that may be originated from distinct biological pathways but that have in common the ability to reach the extracellular environment (van Niel et al., 2018; Rizzo et al., 2020b). Bacteria, fungi, plants, and animals can produce vesicles that can be found intra- or extracellularly, which is an evolutionary capacity conserved among all the kingdoms of life (Deatherage and Cookson, 2012; Brown et al., 2015; van Niel et al., 2018). These structures are highly heterogeneous, especially in their biogenesis, size, and the content they carry (Willms et al., 2018; recently revised by Rizzo et al. (2021)).

Pathogenic fungal EVs were first described in 2007 for *Cryptococcus neoformans* by Rodrigues and co-workers and have gained massive attention since that (Rodrigues et al., 2007). The first characterization of EVs produced by melanized fungi was obtained from *Alternaria infectoria* (*Dematiaceae*), an environmental filamentous fungus involved with crop infestations that can also infect immunocompromised individuals (Silva et al., 2014). Only 6 years later, in 2020, human pathogenic black fungi’s EVs were reported for *Exophiala dermatitidis* (*Herpotrichiellaceae*), a clinically important extremophilic black yeast (Lavrin et al., 2020). To our knowledge, this present work is the first to collect the data on EVs produced by species of the genus *Fonsecaea*.

Once fungal EVs are involved with cell-to-cell communication and are associated with fungal pathogenicity mechanisms, those structures are important elements for the host-pathogen interaction during CBM (Rodrigues and Nimrichter, 2022). In the present work, we characterized for the first time the EVs produced by the pathogenic species *F. pedrosoi* and *F. nubica* in comparison to the EVs obtained from the environmental species *F. erecta*. We characterized their size, morphology, and ergosterol/protein content and their ability to modulate pro- and anti-inflammatory cytokine and nitric oxide production by bone marrow-derived macrophages (BMDMs).

## Materials and Methods

### Fungal Strains and Culture Conditions

Conidia, hyphae, and MCs from *F. nubica* (kindly provided by Dr. Márcia Melhem from Adolfo Lutz Institution, Brazil – strain IAL4), *F. pedrosoi* (strain CBS271.37), and *F. erecta erecta* (strain CBS 125763) provided by Professor Vânia A. Vicente from Federal University of Paraná, Brazil, were used for comparison. The first two strains are clinical isolates (pathogens), while the last one was isolated from plant debris (environmental). The isolates were grown in Potato Dextrose Broth (PDB) medium pH 5.6 under 120 rpm for 21 days. Then, fungal propagules (2×10^7^/mL) were extracted and inoculated into mice hindpaw footpads (50 µL/footpad) to enhance fungal virulence. After 21 days, the animals were euthanized by CO_2_ exposure. The hindpaw plantar tissues were surgically removed, homogenized, and spread on Sabouraud Dextrose Agar medium (SDA). The recovered colonies were used as inoculum under the following experimental conditions.

To obtain saprophytic forms of the fungi, the strains were grown in PDB pH 5.6 and Butterfield and Johnson (BFJ) pH 6.5 media, both at 30°C under 120 rpm for 21 and 25 days, respectively. To obtain MCs, the strains were grown in BFJ pH 2.5 supplemented with 800 µM of DL-propranolol (Thermo Fischer Scientific - Waltham, Massachusetts, EUA) at 30°C under 120 rpm and 40 days, adapted from Alviano and colleagues’ protocol (Alviano et al., 1992). PDB is not a chemically defined medium. It comprises 100 g/L of filtered *Solanum tuberosum*, 10 g/L of dextrose (Thermo Fisher Scientific), and 25 µg/mL of chloramphenicol, and here is named as rich medium (RM). BFJ medium comprises 1.5 g/L of NH_4_NO_3_; 5 mL/L of glycerol 100%; 0.1 g/L of MgSO_4_; 1.8 g/L of KH_2_PO_4_; 0.05 µg/mL of biotin; and 0.1 µg/mL of thiamine and in this work was considered a minimum medium (MM).

### EVs Isolation

The EVs isolation protocol was adapted from Rodrigues and co-workers (Rodrigues et al., 2007). After 21, 25, and 40 days as previously described for each growth condition, the fungal cells were separated from the supernatant through centrifugation and filtered in 0.80 μm and 0.45 μm pore size paper membranes to remove fungal debris. Then, the supernatant was filtered in the ultrafiltration Amicon system with a 100 kDa membrane (Merck Millipore) and subject to ultracentrifugation at 100,000 *×* g at 4°C for 1 h. The pellet was suspended in 300 µL of phosphate-buffered saline (PBS) supplemented with gentamicin (10 mg/mL) and stored at -20°C. The EVs were independently isolated two times from each experimental condition and were used for experiments within 1 month after obtaining them. Before the experiments, the EVs’ suspensions were homogenized with two cycles of 30 min with the Quimis Ultrassonic Bath at the frequency of 50KHz.

### EVs’ Ergosterol and Protein Quantification

The ergosterol was quantified by fluorometric assays utilizing the Amplex Red Cholesterol assay kit (Thermo Fisher Scientific) with excitation at 540 nm and emission at 590 nm. Colorimetric assays were used to quantify the protein content by the Micro BCA assay kit (Thermo Fisher Scientific) at 562 nm. Both analyses were done according to the manufacturer’s instructions.

### Dynamic Light Scattering

The hydrodynamic diameter (nanometers) and polydispersion of the EVs were quantified by Dynamic Light Scattering (DLS) with the “Zetasizer Nano ZS,” which exhibits size intensity peeks (%).

### Bright Field Microscopy

Muriform cells were observed in an inverted epifluorescence microscope Zeiss Axio Observer Z1 equipped with a 40× objective and cooled CCD camera. Bright-field images were collected with the Zeiss ZEN software. The resulting images were processed with the software ImageJ Version 2.1.0/2.53c. No manipulations were made in the pictures.

### Transmission Electron Microscopy

Characterization of EVs was performed by Transmission Electron Microscopy (TEM). Between 3 and 5 mL of the EVs were ultra-centrifuged at 100,000 *×* g at 4°C for 1 h and resuspended in 50 µL of Karnovsky’s Fixative composed of 2% paraformaldehyde and 2% glutaraldehyde diluted in PBS at 0.1 M for at least 24 h. Samples were negatively stained with uranyl acetate and then observed with the Jeol JEM-1011 transmission electron microscope at 80 kV.

### Mice Ethical Agreement

All experimental animal procedures were carried out with C57BL/6 male mice between 8 and 10 weeks of age. The procedures were approved by the Animal Ethics Committee of the University of Brasília (UnBDoc n°. 46/2017) and conducted according to the Brazilian Council for the Control of Animal Experimentation (CONCEA) guidelines.

### Bone Marrow-Derived Macrophages Differentiation

Bone Marrow-Derived Macrophages (BMDMs) were obtained from cultures of hematopoietic stem cells isolated by mice femoral lavage and differentiated using GM-CSF, as previously described (Lutz et al., 1999). BMDMs were resuspended in RPMI 1640 (Gibco-Thermo Fisher Scientific) medium supplemented with 2% Fetal Bovine Serum and 80 µg/mL of gentamicin and incubated at 1×10^6^ cells/mL in 96-well plates at 37°C and 5% CO_2_. After differentiation, BMDMs were washed with RPMI 1640 to remove FBS debris from the supernant and were resuspended in non-supplemented RPMI 1640 for experiments.

### EVs Interaction With BMDMs

BMDMs were stimulated or not with 500 ng/mL of lipopolysaccharide (LPS – Sigma-Aldrich) for 4 h to promote inflammasome priming. Then, according to the number of vesicles obtained from each condition, 80 ng/mL of EVs were determined to be used in the experiments and were added for 20 h, followed by stimulation with 20 μM nigericin for 40 min to provide the second signal for inflammasome activation. Control groups included conditions without LPS and/or nigericin. After interaction, supernatants were collected and stored at -20 °C for further cytokine quantification by enzyme-linked immunosorbent assay (ELISA).

### Cytokine Quantification

Cytokine production was quantified from cell-free supernatants by ELISA. Tumor necrosis factor (TNF), IL-1β, and IL-10 were measured according to the Ready-Set-Go! Kit’s (Thermo Fisher Scientific) specifications. The data were calculated based on a standard curve provided by the commercial kit, and production levels were expressed as pg/mL plus the standard deviation (SD) of two or three independent experiments conducted with technical triplicates each.

### Nitric Oxide Quantification

The production of nitric oxide (NO) was indirectly measured by the release of nitrite ( $NO_{2}^{-}$ ) in the cell-free supernatants cultures of BMDMs by Griess microplate assay, as described by Pick and Mizel (1981). To evaluate the levels of $NO_{2}^{-}$ in the supernatants, BMDMs were incubated with 80 ng/mL of EVs for 20 h in the presence or not of LPS (500 ng/mL - Sigma-Aldrich). One hundred microliters of the supernatants were incubated with an equal volume of the Griess reagent (NEED – N-ethyl-N-diamine 0.1% and sulfanilamide 1% in H_3_PO_4_ 5%) at room temperature, and the absorbance was evaluated at 540 nm using a microtiter plate spectrophotometer. The $NO_{2}^{-}$ concentration was determined utilizing a standard curve ranging from 1 to 200 μM $NO_{2}^{-}$ .

### Statistical Analysis

The data presented in this work are represented by three technical replicates of at least two independent biological replicates. Statistical analyses were made with GraphPad Prism v. 8.0 using one-way analysis of variance (ANOVA) to compare results. Values of less than 0.05 were considered significant, demonstrated with *, when *p* < 0.05; with **, when *p* < 0.01; with *** when *p* < 0.001; and with **** when *p* < 0.0001. Error bars are standard errors of the mean (SEM). For standardization, the data were log-transformed and median centered.

## Results

### *F. pedrosoi*, *F. nubica*, and *F. erecta* Are Able to Produce MC Formation *In Vitro*

**Figure 1:**
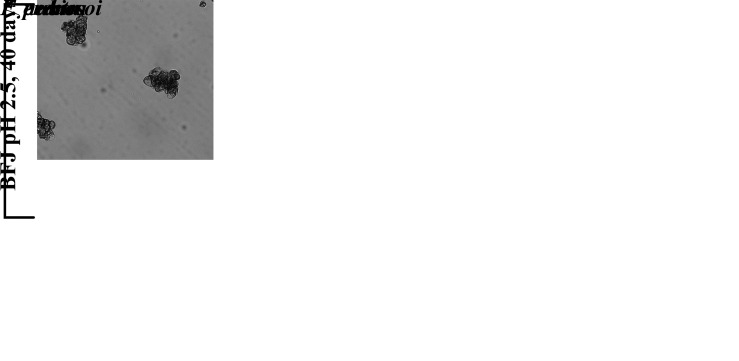
*F. pedrosoi*, *F. nubica*, and *F. erecta* can induce different types of muriform cell formation *in vitro*. Representative images of muriform cells produced by *F. pedrosoi, F. nubica, and F. erecta*. Micrographs were acquired with the inverted epifluorescence microscope Zeiss Axio Observer Z1 with a 40x objective, and bright-field images were processed and analyzed by Zeiss ZEN and ImageJ software, respectively. Scale bars: 10 µm. The results presented in this work refer to a representative triplicate group experiment of at least two independent assays.

To assess if *F. pedrosoi*, *F. nubica*, and *F. erecta* could promote MC development *in vitro*, slides of 40-day-old cultures in BFJ medium pH 2.5 were analyzed by bright field microscopy. We observed that all three *Fonsecaea* species developed MCs. However, while *F. pedrosoi* and *F. nubica* were morphologically close, displaying clumps of dark thick-walled enlarged round cells, indicative of meristematic growth; *F. erecta* induced smaller and less well-developed MCs associated with hyphae formation (**Figure 1**). The inability of *F. erecta* to produce classic MCs morphologically similar to *F. pedrosoi* and *F. nubica* is probably because it is not a human pathogenic species.

### The growth Conditions of the Media Modulate EVs Ergosterol and Protein Content, Size, and Morphology

**Figure 2:**
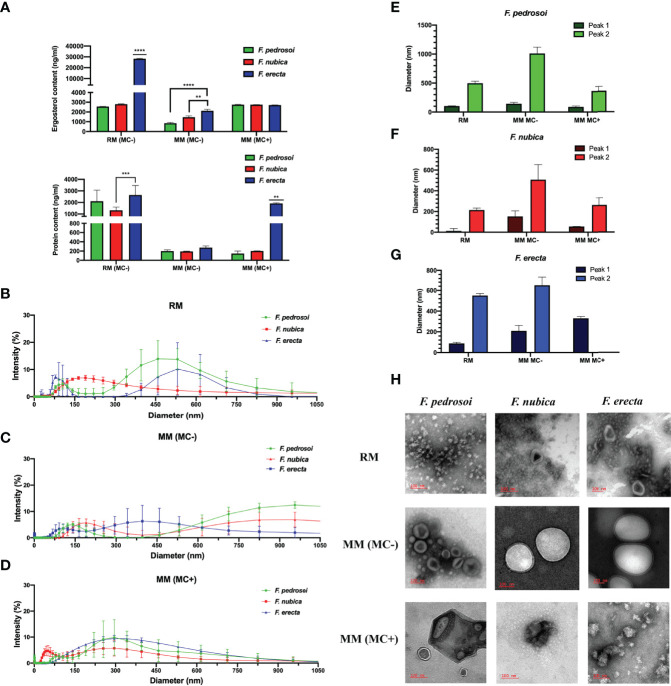
*F. pedrosoi*, *F. nubica*, and *F. erecta* can produce extracellular vesicles (EVs), which are affected by growth conditions. EVs were isolated from *F. pedrosoi* (green), *F. nubica* (red), and *F. erecta* (blue). Ergosterol and protein content **(A)** of the EVs were measured by Amplex Red Cholesterol assay kit and Micro BCA Protein kit assay, respectively. Dimensional analyses by Dynamic Light Scattering (DLS) of EVs isolated from rich medium **(B)**, minimum medium without muriform cells (MCs) **(C)**, and minimum medium with MCs **(D)**. Average diameter peaks by DLS plotted for *F. pedrosoi* **(E)**, *F. nubica* **(F)**, *and F. erecta* **(G)**. Transmission Electron Microscopy (TEM) imaging was used to assess the EVs’ morphology and distribution in all conditions **(H)**. Scale bars: 100nm. For all plots, ** indicates *p* < 0.01, *** indicates *p* < 0.001, **** indicates *p* < 0.0001 according to one-way analysis of variance (ANOVA). The results presented in this work refer to a representative triplicate group experiment of at least two independent assays.

We first analyzed ergosterol and protein content of the EVs produced by *F. pedrosoi*, *F. nubica*, and *F. erecta* isolated from rich medium (RM), minimum medium (MM) without MCs (MM MC-), and MM with the presence of MCs (MM MC+) (**Figure 2**). In general, the levels of ergosterol production between conditions are fairly similar, even though the EVs isolated from RM and MM (MC+) produced by the three species of *Fonsecaea* seem to have higher ergosterol content than EVs obtained from MM (MC-) conditions, which indicates that mycelial forms of *Fonsecaea* might be able to produce higher levels of EVs in an environment with more nutritional availability (**Figure 2A**). Moreover, *F. erecta* demonstrated a greater capacity to produce ergosterol in all three conditions, especially in RM, indicating that these species probably have a higher capacity to produce EVs or present larger EVs when compared to the pathogenic species (**Figure 2A**). The MCs from *F. pedrosoi* and *F. nubica* produce the same levels of EVs as pathogenic mycelium produced in RM (**Figure 2A**). In regard to protein production, isolated EVs in RM from the three species have higher protein content than EVs obtained from the MM conditions, with or without MC, except for *F. erecta* in MM (MC+) which demonstrated a drastic difference in protein production compared to *F. pedrosoi* and *F. nubica* (**Figure 2B**). Furthermore, EVs isolated from *F. pedrosoi* and *F. nubica* induced higher levels of protein content in RM when compared to ergosterol assessment in the same condition.

Next, we investigated hydrodynamic characteristics and EVs polydispersion by Dynamic Light Scattering (DLS) analyses (**Figures 2B–G**). Overall, the EVs exhibited highly heterogeneous patterns across species and growth conditions. In general, the EVs diameter for *Fonsecaea* spp. ranged from 10 to 150 nm, 200 to 600 nm, and 650 to 1500 nm. When obtained from MCs-containing MM, it is observed that the EVs were more homogenous in size, exhibiting diameters mainly from 50 to 500 nm (**Figure 2D**). In contrast, EVs isolated from MCs-free MM were more heterogeneous, encompassing two main peaks: small (50 to 250 nm) and large (<600 nm) diameters throughout all three species (**Figure 2C**). Moreover, the EVs obtained from RM show intermediate size patterns compared to both MM conditions (**Figure 2B**).

When analyzed among species (**Figures 2E–G**), there are two prominent intensity peaks for EVs produced by *F. pedrosoi* in RM: one between 50-150 nm and another one between 300-600 nm; in MM (MC-) two peeks: between 50-250 nm and 550-1000 nm; and in MM (MC+) one peak: 50-500 nm. For EVs produced by *F. nubica* in RM: one main peak 100-500 nm; in MM (MC-) two peeks: between 50-150 nm and 200-600 nm; and in MM (MC+) two peeks: between 50-100 nm and 150-450 nm. Furthermore, EVs produced by *F. erecta* in RM have two distinct peaks: between 0-150 nm and 400-700 nm; in MM (MC-) two peeks: between 100-300 nm and larger than 600 nm; in MM (MC+) one peek: between 100-600 nm.

Similar to the results found by DLS, Transmission Electron Microscopy (TEM) allowed the observation of highly heterogenous EVs concerning their size and morphology when compared both by species and growth conditions (**Figure 2H**). In RM, the EVs are more abundant and have diverse morphological aspects with smaller vesicles. *F. nubica* and *F. erecta* exhibited morphologically similar EVs but with heterogeneous diameters when isolated from MM without MCs. In contrast, EVs from *F. pedrosoi* tend to be more diverse in shape and size. When obtained from MM in the presence of MCs, EVs have different shapes with smaller sizes. In general, TEM images indicate that EVs tend to agglomerate in nearly all culture media.

### EVs From *F. pedrosoi* and *F. nubica* Isolated From the Rich Medium Trigger TNF Production by BMDMs

**Figure 3:**
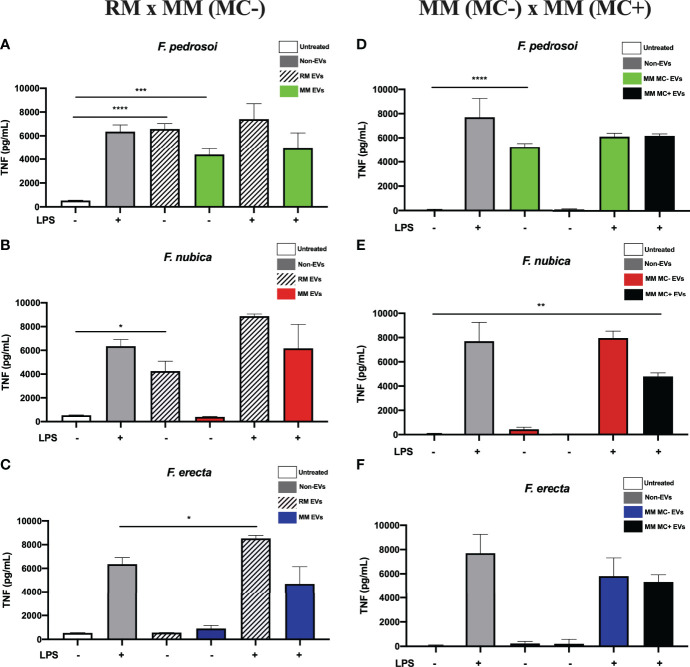
EVs from *F. pedrosoi* and *F. nubica* isolated from the rich medium can promote TNF production by bone-marrow-derived macrophages (BMDMs). MM MC- stands for minimum medium without muriform cells and MM MC+ for minimum medium in the presence of muriform cells. EVs (80 ng/mL of sterol content) from *F. pedrosoi* **(A, D)**, *F. nubica* **(B, E)**, and *F. erecta* **(C, F)** grown in RM, MM MC-, and MM MC+ were incubated with 1x10^6^ BMDMs in the presence or absence of lipopolysaccharide (LPS, 500 ng/mL) for 24h. Cytokine production was assessed by enzyme-linked immunosorbent assay (ELISA). For all plots, * indicates *p* < 0.05, ** indicates *p* < 0.01, *** indicates *p* < 0.001, **** indicates *p* < 0.0001 compared to negative and positive controls according to one-way analysis of variance (ANOVA). The results presented in this work refer to a representative triplicate group experiment of at least two independent assays.

To assess if the EVs can induce a pro-inflammatory response by modulating TNF secretion, BMDMs were co-cultured or not with LPS and 80 ng/mL of EV sterol content produced by morphotypes of *F. pedrosoi*, *F. nubica*, and *F. erecta* for 24 h (**Figure 3**). The EVs from conidia and hyphae of the pathogenic species of *Fonsecaea* isolated from highly nutritional medium (RM) could promote TNF production without LPS. Additionally, EVs produced by *F. nubica* and *F. erecta* grown in RM exerted an additive effect on TNF secretion elicited by LPS. Only *F. pedrosoi’s* EVs elicited TNF levels when isolated from MM (MC-) (**Figures 3A, B**).

Furthermore, EVs produced in MM (MC+) by *F. pedrosoi* and *F. nubica* were not able to promote TNF secretion by BMDMs, indicating they cannot stimulate a pro-inflammatory response alone (**Figures 3D–F**). Co-administration of EVs derived from *F. nubica* but not *F. pedrosoi* or *F. erecta* with LPS altered LPS-induced TNF levels.

### Growth Conditions Influence the Ability of *Fonsecaea* EVs to Modulate IL-1β Production by BMDMs

**Figure 4:**
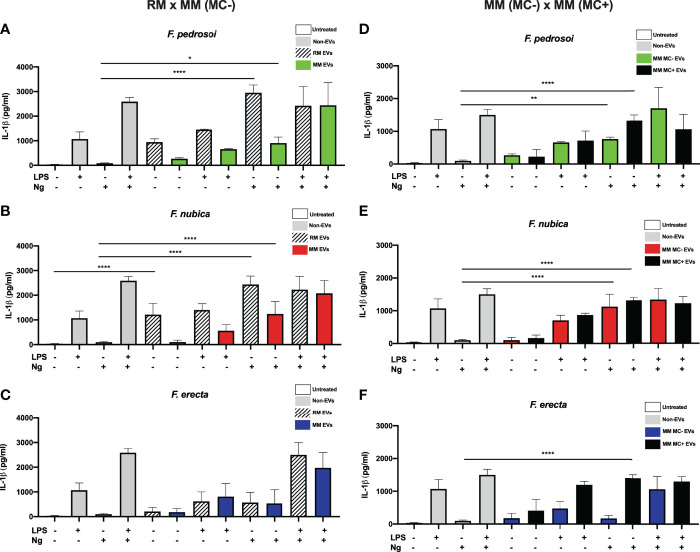
EVs isolated from the rich medium trigger IL-1β production possibly acting as the first signal of inflammasome activation. MM MC- stands for minimum medium without muriform cells and MM MC+ for minimum medium in the presence of muriform cells. EVs (80 ng/mL of sterol content) from *F. pedrosoi* **(A, D)**, *F. nubica* **(B, E)**, and *F. erecta* **(C, F)** grown in RM, MM MC-, and MM MC+ were incubated with 1x10^6^ BMDMs in the presence or absence of lipopolysaccharide (LPS, 500 ng/mL) and/or nigericin (Ng, 20 μM) for 24h. IL-1β secretion was analyzed by enzyme-linked immunosorbent assay (ELISA). For all plots, * indicates *p* < 0.05, ** indicates *p* < 0.01, **** indicates *p* < 0.0001 compared to negative and positive controls according to one-way analysis of variance (ANOVA). The results presented in this work refer to a representative triplicate group experiment of at least two independent assays.

To verify if the EVs could modulate IL-1β secretion, an important product of inflammasome activation, BMDMs were incubated with 80 ng/mL of EV sterol content produced by *F. pedrosoi, F. nubica*, and *F. erecta* isolated from RM, MM (MC-), and MM (MC+) medium. The co-cultures were stimulated with either LPS (a classic first signal inducer) and/or nigericin (a second signal inducer) (**Figure 4**).

The EVs produced by *F. pedrosoi* and *F. nubica* in RM promoted IL-1β secretion in levels comparable to the controls non-stimulated or stimulated with LPS. However, co-addition of nigericin but not LPS enhanced IL-1β secretion (**Figures 4A, B**). These results indicate that these vesicles might act as the first signal in inflammasome activation. The EVs produced in MM (MC-) and MM (MC+) showed a similar modulation pattern of IL-1β release to RM EVs (**Figures 4A, B, D, E**). However, EVs obtained from RM promoted higher levels of IL-1β secretion in the same conditions compared to MM conditions, reaching levels comparable to LPS plus nigericin positive control (**Figures 4A, B, D, E**). In contrast, *F. erecta* RM and MM (MC-) EVs cannot act as the first or second signal for inflammasome activation. However, when produced in MM (MC+), *F. erecta* EVs can act as the first signal like *F. pedrosoi* and *F. nubica* EVs (**Figures 4C, F**).

### The EVs Produced by *Fonsecaea* Pathogenic Species Promote Anti-Inflammatory Response by BMDMs

**Figure 5:**
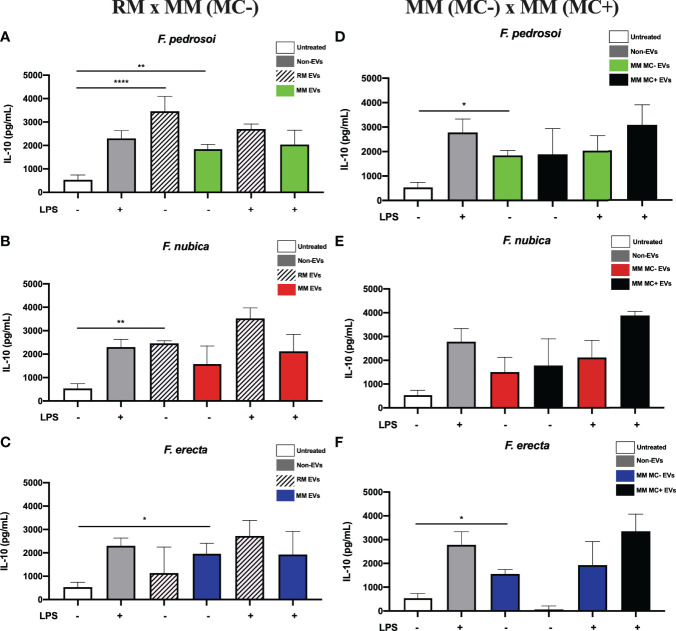
EVs from *F. pedrosoi* and *F. nubica* isolated from the rich medium promote IL-10 production by bone-marrow-derived macrophages (BMDMs). MM MC- stands for minimum medium without muriform cells and MM MC+ for minimum medium in the presence of muriform cells. EVs (80 ng/mL of sterol content) from *F. pedrosoi* **(A, D)**, *F. nubica* **(B, E)**, and *F. erecta* **(C, F)** grown in RM, MM MC-, and MM MC+ were incubated with 1x10^6^ BMDMs in the presence or absence of lipopolysaccharide (LPS, 500 ng/mL) for 24h. IL-10 production was quantified by enzyme-linked immunosorbent assay (ELISA). For all plots, * indicates *p* < 0.05, ** indicates *p* < 0.01, **** indicates *p* < 0.0001 compared to negative and positive controls according to one-way analysis of variance (ANOVA). The results presented in this work refer to a representative triplicate group experiment of at least two independent assays.

IL-10 is an important anti-inflammatory cytokine to combat pathogens and prevent tissue damage. This cytokine can generally hamper Th1 cells’ function, as well as the differentiation of macrophages, NK cells, and DC cells. Since CBM is a chronic disease with marked extensive tissue damage, it is important to assess if *Fonsecaea* EVs have a role in the production of IL-10. Thus, after examining pro-inflammatory cytokine secretion (TNF and IL-1β), we verified whether the EVs produced by *Fonsecaea* spp. can also stimulate an anti-inflammatory response by BMDMs. Interestingly, only EVs produced by *F. pedrosoi* and *F. nubica* in RM boosted the cytokine levels (**Figures 5A, B**). Of note, addition of LPS did not significantly increase IL-10 (**Figures 5A, B**). The EVs produced by *F. pedrosoi and F. erecta* in MM (MC-) also induced IL-10 secretion; however, the *F. nubica* did not show the same pattern (**Figures 5A–C**). The EVs obtained from MM (MC+) produced by all three species cannot increase the IL-10 release (**Figures 5D–F**).

### EVs of *F. pedrosoi and F. nubica*, Obtained From RM, But Not From MM Conditions, Were Able to Promote Nitric Oxide Production by BMDMs

**Figure 6:**
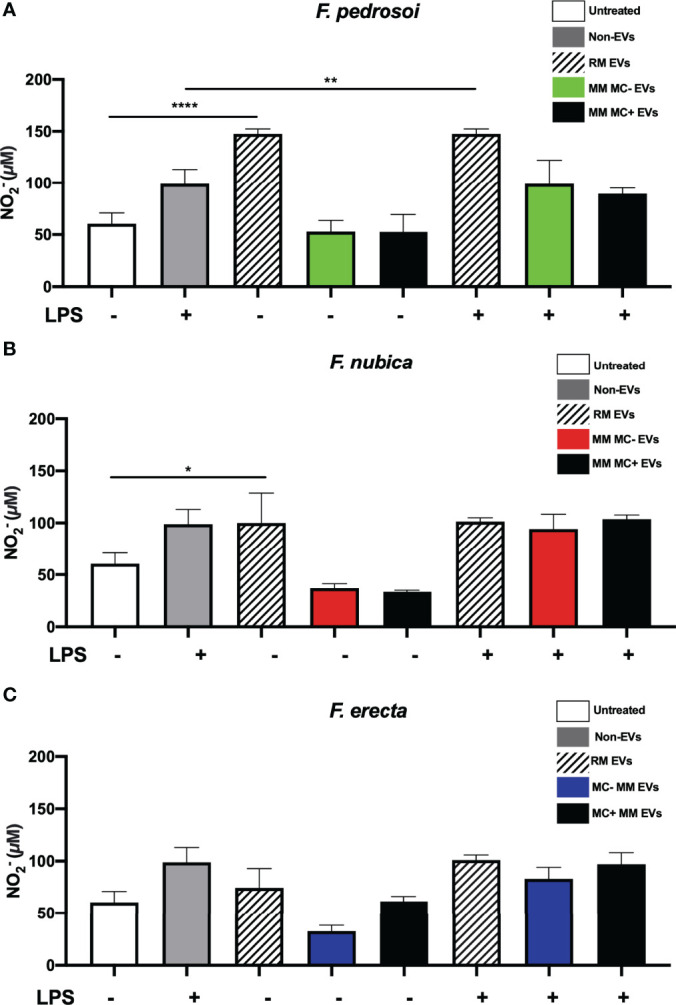
$NO_{2}^{-}$ production by BMDMs is triggered by EVs of *F*. *pedrosoi* and *F. nubica* obtained from RM, but not from MM conditions. MM MC- stands for minimum medium without muriform cells and MM MC+ for minimum medium in the presence of muriform cells. Evs (80 ng/mL of sterol content) from *F. pedrosoi* **(A)**, *F. nubica* **(B)**, and *F. erecta* **(C)** grown in RM, MM MC-, and MM MC+ were incubated with 1x10^6^ BMDMs in the presence or absence of lipopolysaccharide (LPS, 500 ng/mL) for 24h following assessment of $NO_{2}^{-}$ production detected by Griess microplate assay. For all plots, * indicates *p* < 0.05, ** indicates *p* < 0.01, **** indicates *p* < 0.0001 compared to negative and positive control according to one-way analysis of variance (ANOVA). The results presented in this work refer to a representative triplicate group experiment of at least two independent assays.

Finally, we investigated the capacity of the EVs to modulate nitric oxide (NO) production by BMDMs, an important antimicrobial molecule released by phagocytes against different fungal species, including *Fonsecaea* spp. (**Figure 6**). Only the EVs isolated from RM were able to raise the levels of $NO_{2}^{-}$ , a NO byproduct, in the cell supernatant in the absence of LPS. In contrast, the EVs obtained from both MM conditions did not alter $NO_{2}^{-}$ levels (**Figures 6A–C**). Moreover, the EVs produced by *F. pedrosoi* in RM induced higher amounts of $NO_{2}^{-}$ independent of LPS stimulation compared to the EVs from *F. nubica* and *F. erecta* in the same condition (**Figure 6A**).

## Discussion

In the present work, we described and characterized for the first time EVs produced by *F. pedrosoi*, *F. nubica*, and *F. erecta*. *F. pedrosoi* and *F. nubica* species are etiologic agents of chromoblastomycosis (CBM), whereas *F. erecta* is an environmental fungus (Najafzadeh et al., 2010; Vicente et al., 2014; Queiroz-Telles et al., 2017; You et al., 2019). Fungal EVs have a role not only in fungal metabolism but also in fungal pathogenicity and survival inside the host (Rodrigues et al., 2008; Bielska et al., 2018; Ikeda et al., 2018; Zhao et al., 2019; Rizzo et al., 2020a). Although there is growing evidence about the virulence roles of these vesicles, especially for yeast-like fungi such as *C. albicans*, *C. neoformans*, *Saccharomyces cerevisiae*, and *Histoplasma capsulatum*, little is known for filamentous and melanized fungi (Albuquerque et al., 2008; Rodrigues et al., 2008; Oliveira et al., 2010a; Kabani and Melki, 2015; Baltazar et al., 2018; Zhao et al., 2019; Cleare et al., 2020).

The influence of factors such as growth conditions, cell morphology, and fungal strains used in experiments appears to be important in the modulation of EVs biogenesis, size, cargo, and function, although how these factors affect fungal EVs production remains poorly understood (Cleare et al., 2020; Marina et al., 2020; Rizzo et al., 2020a). Since morphological transition and MC establishment during CBM is crucial for *Fonsecaea* survival and pathogenicity, understanding how nutritional conditions and growth factors interfere with EVs characteristics and host-cell communication might grant new insights regarding the roles of extracellular components produced by *Fonsecaea* spp. (de Castro et al., 2017; Siqueira et al., 2017; Dong et al., 2018; Siqueira et al., 2020). For the comparative characterization of the EVs, three experimental conditions were utilized to isolate the vesicles: non-defined rich medium with mainly conidia and hyphae (mycelium) formation (RM); defined minimum medium with mostly hyphae and few conidia without MCs formation (MM MC-); and defined minimum medium with predominantly MCs and hyphae formation (MM MC+).

The first interesting observation is that the pathogenic species *F. pedrosoi* and *F. nubica* induced MC formation, although *F. erecta* did not display the classical described globular and clump morphology as observed for *F. pedrosoi* and *F. nubica* (Mcginnis, 1938; Alviano et al., 1992). The ability of an environmental species to produce MCs, a type of cell strongly related to CBM, might be due to the nature of infection by species of the genus *Fonsecaea* demonstrated to be accidental and not intentional (de Hoog et al., 2004; Fornari et al., 2018). Vicente et al. (2017) have previously investigated the pathogenicity of *F. pedrosoi, F. monophora*, and *F. erecta*. By using *G. mellonella’s* larvae as a model of infection, they observed that *F. monophora* (pathogenic) and *F. erecta* similarly led to a decrease in the life span of the larvae when compared to *F. pedrosoi*. It was also demonstrated that BALB/c mice infection by *F. pedrosoi* and *F. erecta* exhibited similar fungal load levels and kinetics with enhanced production of pro-inflammatory cytokines in the tissue such as TNF, IL-1β, IL-6, and MCP-1 (Vicente et al., 2017). In this sense, non-pathogenic species of *Fonsecaea* exhibit infecting animal tissues capacity.

Furthermore, it has also been demonstrated that *F. erecta* presented higher virulent capacity in *Tenebrio molitor* larvae model than the pathogenic species (Fornari et al., 2018). Initially, the infection by *F. erecta* did not trigger the host immune defense system which might be explained by the fact that the primary host of *F. erecta* infections is plants and not animals; hence, the extracellular components produced by this fungus are probably involved with environmental interactions. Thus, the poor capacity of the EVs produced by *F. erecta* to stimulate cytokine production by macrophages might be related to the fact that these extracellular structures are not originally adapted to the molecular machinery of animal hosts.

Lipids and proteins are fundamental molecular components found in a vast array of fungal EVs that can be used as indicators of the levels of vesicles production (Oliveira et al., 2010b; Baltazar et al., 2018; Zhao et al., 2019; Cleare et al., 2020; Lavrin et al., 2020; Marina et al., 2020). In the present work, it was observed that *Fonsecaea* EVs isolated from the highly nutritional medium led to enhanced levels of ergosterol and protein content compared to EVs obtained from both MM conditions. When the fungal species were compared, *F. erecta* displayed the highest amounts of proteins and sterols in nearly all conditions, indicating that this fungus might produce higher numbers of EVs compared to *F. pedrosoi* and *F. nubica* (**Figure 2A**). The aptness to generate highest amount of EVs might be because that *F. erecta* has initially been obtained from the environment and thus, has a predisposition to grow in conditions similar to experimental conditions. Nutritional availability of the culture medium has been shown to modulate the lipid and protein content of EVs produced by *C. neoformans* and *H. capsulatum* (Cleare et al., 2020; Marina et al., 2020). EVs produced by *C. neoformans* in MM (Sabourad Dextrose Broth 50%) presented elevated levels of ergosterol/protein ratio when compared to the vesicles isolated from RM (Sabourad Dextrose Broth 100%) (Marina et al., 2020). For *H. capsulatum*, three conditions were used to compare EVs features, in which was observed higher ergosterol and protein content in vesicles produced in HAM’s F12 media (Cleare et al., 2020). Also, a comparison between fungi from the same genus, *Candida glabrata*, *C. parapsilosis*, and *C. tropicalis*, showed heterogeneous levels of protein and phospholipid content (Karkowska-Kuleta et al., 2020), reiterating that not only nutritional availability but also fungal species and strain can modulate EVs biogenesis, accordingly to the results presented in this research.

Size analyses of EVs are indicators of their biogenesis’s pathways and cargo (Wolf et al., 2015; de Oliveira et al., 2020; Rizzo et al., 2020b; Rizzo et al., 2021). Overall, for *Fonsecaea* EVs, three main populations were identified: (a) small EVs ranging between 10-150 nm; (b) intermediate EVs ranging between 200-600 nm; (c) and large EVs ranging between 650-1500 nm. The EVs bigger than 800 nm probably are vesicles’ agglomerates, as observed through TEM (**Figure 2F**) that cannot be read as individual components by DLS. The DLS results for *F. pedrosoi*, *F. nubic*a, and *F. erecta* EVs indicate that these structures have similar diameter variations, as encountered for *A. fumigatus*, *A. flavus*, *C. albicans*, and *H. capsulatum* (Albuquerque et al., 2008; Vargas et al., 2015; Souza et al., 2019; Brauer et al., 2020). However, Nano Tracking Analysis (NTA) assays must be done in the future to confirm with more precision the diameter of the EVs produced by *Fonsecaea* spp. TEM imaging allowed us to observe the EVs’ morphology and distribution (**Figure 2F**). Most of the EVs observed are similar to previous descriptions, especially for *A. infectoria*, *C. albicans*, *C. glabrata*, *C. parapsilosis, E. dermatitis*, and *Pichia fermentans* (Silva et al., 2014; Leone et al., 2017; Karkowska-Kuleta et al., 2020; Lavrin et al, 2020; Vargas et al., 2020). The EVs of *F. nubica* and *F. erecta* isolated from MM MC- are morphologically similar to the *P. fermentans* EVs, exhibiting extremely rounded shape structures, even though *Fonsecaea* EVs seem more contrasted, probably because of melanin released to the extracellular environment (Alviano et al., 1991; Farbiarz et al., 1992; Leone et al., 2017). An intriguing observation is the presence of electron-dense hexagonal-shaped components associated with the EVs isolated from *F. pedrosoi* when grown in MM (MC+) (**Figure 2F**). These structures appear to be equivalent in size and shape to electron-dense fragments shed from the cell wall of *F. pedrosoi* MCs, as reported by Franzen et al. (2006), a type of event that has previously been described (Alviano et al., 1991; Farbiarz et al., 1992). Since melanin, β-1,3-glucan, and α-1,3-glucan are major components of the *Fonsecaea* cell wall and can be recognized by the host immune cells, more detailed analyses should be done to assess the importance of these structures in the host-cell communication during CBM.

Many studies about fungal EVs have indicated that these structures can modulate cytokine production by macrophages and/or dendritic cells, such as for *C. neoformans* (Oliveira et al., 2010a; Marina et al., 2020), *C. abicans* (Vargas et al., 2015; Vargas et al., 2020), *Paracoccidioides brasiliensis* (Silva et al., 2016), *Sporothrix brasiliensis* (Ikeda et al., 2018), *A. fumigatus* (Souza et al., 2019) and *A. flavus* (Brauer et al., 2020). In the present work, it was demonstrated that only the EVs produced by the pathogenic species of the genus *Fonsecaea* obtained from RM could stimulate TNF and IL-10 production by BMDMs, whereas the EVs isolated from both MM conditions were not able to promote these patterns (**Figures 3**, **5**). Moreover, EVs produced in RM and MM (MC-) by *F. pedrosoi* and *F. nubica*, and in MM (MC+) by the three species, were able to elicit significant levels of IL-1β when administered with only nigericin, which indicates that, in those conditions, the EVs might act as the first signal in inflammasome activation (**Figure 4**). For EVs of the filamentous fungus *A. fumigatus*, it was evidenced that their vesicles carried not only proteins with virulent capacities, like allergens, chaperones, superoxide dismutase, nucleoside diphosphatase kinase, and more, but also could enhance fungal clearance and promote a pro-inflammatory response by macrophages through the production of TNF, IL-1β and CCL2 (Souza et al., 2019). Similar results were found for EVs produced by *A. flavus* which their vesicles activated TNF, NO, IL-6, and IL-1β, promoted enhanced phagocytosis and stimulated M1 polarization by macrophages *in vitro* (Brauer et al., 2020). The fungal EVs’ potential to induce M1 polarization has also been shown for *P. brasiliensis’* EVs that stimulated TNF, IL-6, and IL-12p production by peritoneal macrophages and promoted M1 activation through the expression of iNOS, a classical marker for M1 polarization (Silva et al., 2016). Altogether, these data show that fungal EVs can act as immunomodulatory components in host-fungi interactions and that growth conditions can affect this ability.

Marina et al. (2020) revealed that *C. neoformans* EVs not only modulated cytokine production by bone marrow-derived macrophages and dendritic cells but that these modulator capacities were influenced by medium composition. Furthermore, *C. neoformans* EVs could induce IL-1β only when combined with nigericin, acting as the first signal for inflammasome activation, similar to the results observed in the work here presented. Further analyses of inflammasome signaling pathways, such as the cleavage of pro -IL-1β and production of other cytokines (e.g., IL-18) should aid to comprehend the mechanisms underlying *Fonsecaea* species’ EVs impact on inflammasome activation.

Besides cytokine production, macrophages have another important molecular mechanism against the infection by *Fonsecaea* spp. as observed in CBM, releasing oxygen and nitrogen reactive species, such as NO and H_2_O_2_ (Bocca et al., 2006; Cunha et al., 2010). Fungal EVs have been demonstrated to stimulate NO production by macrophages and/or dendritic cells, which indicates that these structures may enhance fungicidal activity by phagocytes (Oliveira, et al., 2010a; Vargas et al., 2015; Joffe et al., 2016; Silva et al., 2016; Brauer et al., 2020; Marina et al., 2020). The EVs produced by *A. flavus*, *C. albicans*, *C. neoformans*, and *P. brasiliensis* can promote NO secretion, measured by both iNOS expression and nitrite production (Oliveira, et al., 2010a; Vargas et al., 2015; Silva et al., 2016; Brauer et al., 2020; Marina et al., 2020). Moreover, for *A. flavus* and *P. brasiliensis*, the enhanced production of NO after the interaction of macrophages and EVs was related to M1 polarization and increased fungicidal activity (Silva et al., 2016; Brauer et al., 2020). Thus, further investigation about the possible roles for *Fonsecaea* EVs in M1 polarization should be addressed.

In this research, the EVs isolated from a medium composed mainly of conidia and hyphae (RM), in general, produced EVs with higher immunostimulatory capacities than those obtained from MM mainly composed of hyphae or MCs (MM MC- and MM MC+). Interestingly, it has been shown that hyphae and MCs, but not conidia, can induce IL-1β and TNF production by murine macrophages and a human monocytic cell line *in vitro* (de Castro et al., 2017). Hyphae and especially MCs are involved with the chronicity of CBM. In contrast, conidia could not reach the chronic phase of the disease, being rapidly eliminated from the injury in a mice model of infection (Siqueira et al., 2017). Moreover, only the cells mentioned above, but not conidia of *F. pedrosoi*, were able to induce a pro-inflammatory response by macrophages and dendritic cells marked by TNF, IL-1β, IL-6, and MCP-1 production, which corroborates the fact that only the interaction between macrophages and MCs were able to promote the up-regulation of genes related to inflammation, cell migration and fungal recognition (Siqueira et al., 2017). In this sense, the EVs produced by *F. pedrosoi* exhibit a relatively opposite modulatory capacity in stimulating TNF and NO production when the fungi interact directly with macrophages. EVs from culture containing abundant conidia, but not exclusively hyphae nor MCs, were more capable of inducing a pro-inflammatory response. The host-fungi interactions are extremely dynamic and dependent on interactions between the cells and molecules secreted, which can modulate the host immune response pattern in the inflammatory focus.

## Conclusion

In summary, we demonstrated and characterized for the first time EVs produced by pathogenic (*F. pedrosoi* and *F. nubica*) and environmental (*F. erecta*) species of the genus *Fonsecaea.* These structures can modulate pro- and anti-inflammatory cytokine and nitric oxide production by macrophages *in vitro.* This ability is influenced by growth conditions, which suggests that environmental factors encountered by *Fonsecaea* spp. might act as potent regulators of the EVs’ biogenesis and function. Nevertheless, further analysis, especially genomic and proteomic investigations, should be performed to elucidate the possible roles of *Fonsecaea* EVs in host-cell communication far from inflammatory focus and their influence on the cell morphology transition during CBM.

## Data Availability Statement

The original contributions presented in the study are included in the article/Supplementary Material. Further inquiries can be directed to the corresponding author.

## Ethics Statement

The animal study was reviewed and approved by Animal Ethics Committee of the University of Brasília (UnBDoc n°. 46/2017).

## Author Contributions

AB formulated the study. LOL-C, AB, and LF wrote the manuscript. LOL-C, CM, and LC performed the experiments. SB, LF, and LOL-C carried out transmission electron microscopy and optical microscopy. LOL-C, CM, RC, LC, LF, and AB analyzed and interpreted the results. CM, RC, SB, VV, LF, and AB contributed with fungal strains, reagents, and analysis tools. All authors reviewed and approved the manuscript.

## Funding

This work was supported by Fundação de Amparo à Pesquisa do Distrito Federal (193.000.805/2015) and Conselho Nacional de Desenvolvimento Científico e Tecnológico (306515/2019-9). The Doctoral Felloshwip was supported by Coordenação de Aperfeiçoamento de Pessoal de Nível Superior (88882.347193/2019-1).

## Conflict of Interest

The authors declare that the research was conducted in the absence of any commercial or financial relationships that could be construed as a potential conflict of interest.

## Publisher’s Note

All claims expressed in this article are solely those of the authors and do not necessarily represent those of their affiliated organizations, or those of the publisher, the editors and the reviewers. Any product that may be evaluated in this article, or claim that may be made by its manufacturer, is not guaranteed or endorsed by the publisher.
